# Preventing cardiovascular disease in at-risk patients: Results of a pilot behavioural health programme in general practice

**DOI:** 10.1080/13814788.2024.2413106

**Published:** 2024-10-18

**Authors:** John Broughan, Emīls Sietiņš, J. T. Treanor, Ka Yuet Emily Siu, Janis Morrissey, Orla Doyle, Mary Casey, Patricia Fitzpatrick, Geoff McCombe, Walter Cullen

**Affiliations:** a Clinical Research Centre, School of Medicine, University College Dublin, Dublin, Ireland; b School of Medicine, University College Dublin, Dublin, Ireland; c Irish Heart Foundation, Dublin, Ireland; d School of Economics, University College Dublin, Dublin, Ireland; e School of Nursing, Midwifery and Health Systems, University College Dublin, Dublin, Ireland; f School of Public Health, Physiotherapy and Sport Science, University College Dublin, Dublin, Ireland

**Keywords:** Cardiovascular diseases, general practice, health promotion, primary prevention, vulnerable populations

## Abstract

**Background:**

The ‘High-Risk Prevention Programme’ (HRPP) involved a six-week health behaviour change programme based in general practices and aimed to address cardiovascular disease (CVD) risk in disadvantaged Irish communities.

**Objectives:**

This pilot study aimed to establish the HRPP’s likely effectiveness and acceptability to inform the development of a future definitive trial.

**Methods:**

The HRPP was conducted at six general practices in disadvantaged areas in the Ireland East region. Patients with high CVD risk were recruited by participating practices and were allocated to either a General Practice Nurse (GPN) or Health Promotion Professional (HPP) led programme focusing on positive health behavioural change. Baseline and 12-month follow-up data were collected to capture the HRPP’s likely effectiveness in promoting health outcomes and health behavioural change.

**Results:**

The HRPP programme was completed by 270 patients. Out of these 270 patients, 245 (90.74%) completed baseline assessments, and 176 (65.19%) completed follow-up assessments at 12 months. Baseline data indicated a high level of CVD risk among patients and follow-up demonstrated positive change in several areas, especially weight (−1.95 kg, p < 0.001), BMI (−0.72, p < 0.001), exercise during the last week (p<0.001), and consumption of healthy fats in the HPP group (+60%, p< 0.001).

**Conclusion:**

The HRPP was a much-needed pilot intervention, and positive results were seen in both GPN and HPP arms, especially with regards to weight loss, exercise, and dietary improvements. Future definitive trials of the HRPP are likely to be effective and acceptable in terms of combatting these issues among high-risk patients.

## KEY MESSAGES

The High-Risk Prevention Programme promoted positive health/health behaviours among patients with high CVD risk in the participating GP sites.

Its impact in terms of promoting weight loss, exercise, and healthy diet were particularly noteworthy.

Future definitive trials may further demonstrate the initiative’s potential as a public health solution.

## Introduction

Eighteen million people die globally each year because of cardiovascular diseases (CVDs) with 1/3 of these deaths occurring in people under 70 years [1]. Notable CVD risk factors include high blood pressure, cholesterol, diabetes, problematic health behaviours such as poor diet, physical inactivity, and alcohol/tobacco use, as well as environmental factors like air pollution and lower socioeconomic status [2, 3]. Health promotion interventions focusing on primary prevention of CVD in primary care are therefore recommended to tackle the growing prevalence of CVD in communities worldwide [4]. CVD prevention in primary care is well regarded for the emphasis it places on collaborative and integrated working between key stakeholders (e.g. healthcare staff, patients, community representatives) in care planning and delivery, as well as its tendency to prioritise the promotion of general health and wellbeing among patients [5]. However, a ‘one-size-fits-all’ approach to CVD primary prevention is not supported by evidence.

Under the Health Service Executive’s Chronic Disease Management Programme (CDMP), those at high risk of cardiovascular disease or diabetes are entitled to one annual GP visit and one Practice Nurse visit [6]. Such high-risk patients require active management of their risk factors. This is challenging to achieve through an annual visit, particularly for vulnerable people and people in deprived areas. Rather, research indicates that targeted interventions tailored to accommodate and address the unique care needs of specific population sub-groups are required to enact meaningful change [2]. The High-Risk Prevention Programme (HRPP) is an initiative that followed this ethos. In 2020, the Irish Heart Foundation (IHF), in partnership with Ireland’s Health Services Executive (HSE), developed and piloted the HRPP in six general practices (GP) in the Ireland East region, each of which provides care to those communities living in socio-economically deprived areas. The HRPP aimed to action positive health behaviour change among high-risk populations by enhancing patients’ capacity for health self-management. The HRPP complemented Ireland’s recently introduced CDMP. However, to better prevent patients from developing chronic disease, it was decided that a more intensive approach was required, and this approach was piloted in the HRPP.

The HRPP’s scope was large requiring two separate study articles to faithfully document its evaluation. First, a manuscript assessing the feasibility of the HRPP by way of qualitative interviews with staff and patients involved in the programme has been produced for publication. Second, determining the likely effectiveness of the HRPP to ascertain the necessity for and probable contents of a future definitive HRPP trial was also required. This study reports on the latter.

## Methodology

To establish the likely effectiveness of the HRPP intervention, a comprehensive health assessment with patients enrolled in the HRPP was conducted at baseline (i.e. before commencement of the programme) and at 12 months following the programme’s completion. The HRPP’s acceptability was assessed by analysing data on engagement of practices and patients in the programme. Study design was guided by recommendations from the CONSORT 2010 Statement: extension to randomised pilot and feasibility trials’ [7], the ‘Strengthening the Reporting of Observational Studies in Epidemiology (STROBE)’ guidelines [8], and Lancaster and Thabane’s (2019) guidelines for reporting non-randomised pilot and feasibility studies [9].

### Recruitment, setting and participants

In 2020, advertisements requesting expressions of interest from general practices to participate in the HRPP were placed on the IHF website, the ‘Medical Independent’, and the IHF’s social media platforms (Twitter/X, LinkedIn, and Facebook). Seven diverse healthcare practices, representing a range of urban, rural, and mixed locations with varied patient populations and capacities, were chosen to take part in the intervention co-design process. This diverse representation ensured a comprehensive evaluation of the intervention’s potential effectiveness across different settings. One practice, however, declined to participate due to service pressures exacerbated by the COVID-19 pandemic. Patients were included/excluded by participating GP practices if they met the criteria in Table 1*.

**Table 1.** Study inclusion and exclusion criteria.

Inclusion criteria		Exclusion criteria
1	Were 40 years or older.	Were <40 years old.
2	Had a General Medical Services (GMS) scheme or GP visit card. The GMS scheme permits medical card holders to access health services (e.g. primary & secondary care services) participating in the GMS scheme free of charge. GP visit cards allow patients to visit GPs and access select GP services (e.g. blood tests) free of charge.	Did not have a GMS or doctor visit card.
3	Had a high CVD risk including hypertension, high cholesterol, physically inactivity, overweightness / obesity, and Type 2 diabetes patients without access to an alternative lifestyle behaviour change programme, as well as patients with 10-year Q-Risk scores >20%. Patients who smoke were signposted to appropriate smoking cessation services for support. If included in the HRPP with other risks their progress was monitored.	Had an already established CVD (e.g. coronary artery disease, history of stroke / transient ischaemic attack, heart failure) or they had Type 2 diabetes and were attending a different lifestyle behaviour change programme.
4	Were deemed physically and mentally well enough by healthcare professionals to provide informed consent and to participate in the programme.	Were deemed unable to self-manage their health (e.g. patients living with dementia).

* Some included patients did not meet all the criteria (e.g. patients < 40 years of age), but were judged to be suitable for the programme by participating GPs on account of being high-risk.

Patient recruitment was ongoing throughout the project. General practices contacted patients meeting the project’s inclusion criteria inviting them to participate. It was deemed that recruiting approximately 400 patients (GPN arm: 200 patients; HPP arm: 200 patients) would suffice for satisfying statistical power, as well as staffing, time, and recruitment budget considerations. However, patient recruitment was intermittently disrupted by COVID-19 lockdown measures. A pragmatic recruitment approach involving recruiting as many patients as possible in these circumstances was subsequently adopted.

The study received ethical approval from University College Dublin’s Human Research Ethics Committee on 05/03/2021. Informed consent was taken for all participants in the study.

### Procedure

#### Intervention

The HRPP consisted of a six-week health behavioural change consultation program where patients engaged in one-to-one sessions with either a General Practice Nurse (GPN) or a Health Promotion Professional (HPP). Patients attended six sessions, one per week, throughout the program’s duration. Due to COVID-19 lockdown restrictions, a combination of in-person and virtual sessions were conducted intermittently. This 6-week program was held at each of the six participating practices, with three practices offering the GPN intervention and three providing the HPP intervention. Assessments were conducted in person both at baseline and 12 months after completion of the programme. Both the Health Promotion Professional (HPP) and General Practice Nurse (GPN) arms aimed to facilitate positive health behaviour change among patients by prioritising the enhancement of capacity for health self-management [10, 11]. The programme was guided by a patient-centred philosophy whereby patients, in collaboration with GPN or HPP professionals, determined the programme’s aims, objectives, and procedures according to their own personal health goals and living circumstances.

For each patient, the 6-week programme was bookended by two GPN/HPP administered assessments involving routine health checks and surveying of patients’ health behaviours, mental health, health knowledge, and motivation to be healthier. The follow-up assessments were scheduled at 3, 6, and 9 months after the initiation of the HRPP. However, detailed information regarding these follow-ups was not provided by the general practices due to confidentiality reasons. The initial assessment for each patient occurred before their participation in the HRPP, while the second assessment took place 12 months after the completion of the programme. The assessments facilitated clinical monitoring of patients’ health and care utilisation, as well as the ability to establish the HRPP’s likely effectiveness. Additionally, confidential follow-up calls monitoring patients’ progress were made by the GPN and HPP with each patient at 3, 6, and 9 months following programme completion.

#### Study instruments

Data from the baseline and follow-up assessments included patient demographics (e.g. age, gender, country of birth), indicators of physical health status (e.g. Body Mass Index (BMI), blood pressure & cholesterol), and patients’ self-reported answers to questions regarding their health behaviours (e.g. exercise, diet), mental health, health knowledge (e.g. knowledge of health risk factors), and motivation to be healthier. A blend of original, standardised, and modified questionnaires was included in the survey [12–16] (see Table 2). Data illustrating practice and patient engagement with the programme was also collected. These included:

**Table 2.** Items included in patient assessments at 1 and 12 months.

Characteristic		Items
1	Demographics	Age Gender Education level Employment status
2	Physical health	Height Weight BMI Waist circumference Blood pressure On blood pressure treatment Resting heart rate Blood glucose Cholesterol (LDL, HDL, Triglycerides)
3	Health behaviour	Alcohol use (adapted version of the AUDIT-C scale) (10) Physical activity levels (adapted version of the ‘Short form of the International Physical Activity Questionnaire’) (11) Sedentary behaviour (adapted version of the ‘Rapid assessment disuse index [RADI]’) (12) Smoking Medication adherence Dietary habits
4	Mental health	Mental Health and wellbeing (assessed by the ‘Warwick-Edinburgh Mental Well-being Scale’) (13)
5	Health knowledge	CVD / Stroke risk factors (adapted version of the ‘National Health Services Health Check questionnaire’) (14)
6	Behaviour change	'Willingness to change health behaviours’ as opposed to what’s written their currently (CVD / Stroke risk factors (adapted version of the ‘National Health Services Health Check questionnaire’) [13])

*GPN: General Practice Nurse; HPP: Health Promotion Professional; BMI: Body Mass Index; LDL: Low-density lipoprotein; HDL: High-density lipoprotein; CVD: Cardiovascular disease*.

- Number of practices participating in the programme.
- Number of patients recruited per practice.
- Number (%) patients who completed the 6-week programme.
- Number (%) patients who completed baseline and follow-up health assessments.

#### Data analysis

Descriptive and inferential statistics were used to analyse the data. Descriptive statistics included frequency counts, percentages, measures of central tendency and variation (i.e. mean (standard deviation), median (interquartile range), and measurement of absolute differences). Inferential tests examining statistically significant relationships between study variables were also used (i.e. Paired sample t-tests, Wilcoxon signed rank sum tests, McNemar/McNemar-Bowker tests, mixed factorial ANOVA tests, Fisher’s Exact test). To accommodate potential familywise error rate (FWER) issues resulting from multiple comparisons, statistical significance levels for all inferential tests conducted are reported at liberal and conservative alpha levels where applicable (0.05, <0.01 & <0.001). Missing data were managed using data imputation as required. For example, in the health questionnaire, the overall score was calculated by averaging the responses they provided, then scaling it up to a score out of 10.

## Results

### HRPP engagement data

Six practices commenced and completed the HRPP, and 479 patients were invited to participate across all practices. Of these, 304 patients started the programme (recruitment rate = 67%) and 270 completed it (retention rate = 89%) (Table 3).

**Table 3.** Patient engagement in the HRPP 6-week programme.

Professional delivering the intervention Programme	Number of patients invited (n)	Number of patients commence the programme (n)	Number of patients completed the programme (n)	Recruitment rate (%)	Retention rate (%)
GPN	322	178	162	55	91
HPP	157	126	108	80	86
Total	479	304	270	67	89

### Baseline and follow-up participation/sample characteristics

#### Baseline

Out of the 270 patients that completed the 6 week HRPP intervention programme, 245 patients completed the baseline assessment. A GPN intervention was delivered to 146 (59.6%) patients and a HPP intervention to 99 (40.4%). There were 131 (53.5%) females, 110 (44.9%) males, and patients’ mean age was 58 years (SD = 10.06). As can be seen form Table 4, approximately a quarter of patients had third level education and approximately 18% were employed full-time.

**Table 4.** Education and employment details.

Variable	n	%
***Highest degree of education (n = 237)***		
Primary	63	26.6
Secondary	111	46.8
Third level	61	25.7
Other	2	0.8
***Employment (n = 231)***		
Full-time employed	41	17.7
Part-time employed	24	10.4
Unemployed	37	16.0
Caregiver	12	5.2
Homemaker	33	14.3
Student	1	0.4
Other (e.g. retired, disability)	83	35.9

#### Follow-up

Follow up assessment at 12 months was completed by 176 of 245 patients (71.84%). 111 of 146 (76.03%) of patients in the GPN group completed follow-up assessment, and 65/99 (65.7%) patients in the HPP group did so. Patient gender (males = 43.2%, females = 55.1%) and age distributions (mean age = 59.29 years, SD = 10.46) were like those at baseline.

### Findings from health checks and health surveys

Table 5 outlines baseline and follow-up data regarding the continuous clinical and demographic outcome measures for patients who completed both baseline and follow-up assessments only. Means (standard deviations) are reported for baseline statistics. ‘Change from baseline’ scores, as well as p values for statistically significant findings, are reported for follow-up data. At baseline, many patients were not obese (mean weight = 98.41 kg). It is also noteworthy that a good majority expressed a readiness to change their health behaviour (mean = 8.21/10, SD = 1.66).

**Table 5.** Mean (SD) and ‘change from baseline’ figures for clinical and demographic variables at baseline and 12 months follow-up.

		Baseline	Follow-up
*Variable*	*n*	*Mean (SD)*	*Change from baseline*
Weight (kg)	162	98.41 (22.49)	−1.95 (kg)***
BMI	158	35.15 (7.59)	− 0.72***
Waist circumference (cm)	123	114.24 (14.83)	−1.85 (cm)*
Resting heart rate (bpm)	130	77.17 (11.44)	+0.65 (bpm)
Blood glucose (mmol/L)	148	6.21 (2.01)	−0.23 (mmol/L)
LDL (mmol/L)	153	2.89 (1.02)	−0.08 (mmol/L)
HDL (male) (mmol/L)	71	1.26 (0.49)	−0.25(mmol/L)
HDL (female) (mmol/L)	89	1.33 (0.44)	+0.06 (mmol/L)
Triglycerides (mmol/L)	162	2.13 (1.31)	−0.20 (mmol/L)*
Cholesterol	163	5.05 (1.37)	−0.13
Days spent exercising moderately during last week	22	4.41 (0.04)	−0.41(days)
Minutes spent exercising during last week per day	25	78.12 (69.79)	−0.80 (mins)
Knowledge of cardiovascular risk factors (max score =10/10)	168	6.98 (1.20)	−0.042
Mental health and wellbeing (max score = 70, high scores indicated positive mental health & wellbeing)	164	48.04 (10.78)	+3.56**
‘Readiness to change’ (max score = 10)	170	8.16 (1.67)	−0.68*

* *p* < 0.05.

** *p* < 0.01.

*** *p* < 0.001 (all are Paired Sample t-tests).

At follow-up, the most noteworthy changes from baseline pertained to weight loss (*p* < 0.001) and reduced BMI (*p* < 0.001). Statistically significant changes indicating improved mental health and wellbeing (*p* < 0.01), and reduced waist circumference (*p* < 0.05) and triglyceride scores (*p* < 0.05) were also evident.

At baseline, high blood pressure was common (*n* = 140, 86.9%) and 121 (61.8%) were receiving blood pressure treatment. Three (1.9%) patients had Type 1 Diabetes and 33 (20.9%) had Type 2 Diabetes. Most patients (*n* = 142, 88.2%) received medication for either high blood pressure, cholesterol, or diabetes. Most patients did not smoke (*n* = 109, 69.9%) or drink alcohol (*n* = 108, 61.4%). 42 (25%) said they exercised moderately within the last week. 165 (93.8%) agreed or strongly agreed that they would like to live a healthier lifestyle.

At follow-up, 41 patients (25.5%) switched from having high stage 2 blood pressure to lower blood pressure categories. The number with Type 2 Diabetes increased by 3.8% because of the intervention’s new testing initiatives (*p* = 0.040). The number receiving medication for high blood pressure, high cholesterol, or diabetes increased by 1.2%. Heavy smokers (*n* = 5, 3.2%) were smoking less (*p* = 0.003). There was no meaningful change in the number who drink alcohol. The proportion of patients exercising moderately within the last week increased from 25% to 39.9% (*n* = 67, *p* < 0.001). Fewer patients reported sitting eight hours or more per day (-8.2%, *p* = 0.030) and fewer ‘Strongly agreed’ that they want to live a healthier lifestyle (-15.0%, *p* = 0.002), perhaps indicating that their motivation declined having already achieved their personal health goals by this point.

#### Findings from analysis of baseline and follow-up dietary behaviour data

Details showing patients’ routine dietary intake at baseline and follow-up are in Table 6. The most notable findings in this regard related to patients’ consumption of snacks or salty foods (-12.4%, *p* < 0.01), deep-fried food or fast foods (-11.2%, *p* < 0.01), vegetables (+7.6^1^%, *p* < 0.05), healthy fats (+31.8%, *p* < 0.01), and fruit (+0.33%, *p* < 0.01).

**Table 6.** Dietary behaviour at baseline and 12 months follow-up.

		Baseline	Follow-up
*Variable*	*n*	*Yes (%)*	*Change from baseline (%)*
Eating snacks or salty food one or more times per day	169	57.4	−12.4^1^**
Eat deep-fried food or fast food 3 or more times per week	170	24.1	−11.2^1^**
Eating meat or poultry 3 or more times per day	170	45.9	+5.3^1^
Drinking fizzy drinks one or more times per day	168	21.4	−5.9^1^
Eating fruit one or more times per day	169	70.4	+12.4^1^
Eating vegetables one or more times per day	171	84.2	+7.6^1^*
Include healthy fats (e.g. oily fish, nuts, seeds) in their diet	170	45.3	+31.8^1^**
***Variable***		***Mean (SD)***	***Mean difference***
Portion(s) of fruit eaten per day	105	2.05 (1.09)	+0.33^2^**
Portion of vegetables eaten per day	139	1.97 (0.83)	+0.18^2^

^1^ McNemar tests.

^2^ Paired sample t-tests.

* *p* < 0.05.

** *p* < 0.01.

*** *p* < 0.001.

### Differences in outcomes between HPP and GPN groups

At follow-up, both GPN (*n* = 111) and HPP (*n* = 65) groups yielded positive outcomes related to improved cardiovascular health. Two statistically significant differences were observed when comparing differences in continuous outcomes between GPN and HPP arms using mixed factorial ANOVA tests. The HPP group showed a larger increase in the average number of daily vegetables consumed (+0.41) compared to the GPN group (+0.06) (p < 0.05). Also, the HPP group demonstrated a mean increase in CVD health risk knowledge scores (+0.37 points) while the GPN group’s scores on this measure decreased by 0.26 points (*p* < 0.01). When comparing differences in categorical outcomes between GPN and HPP arms using Fisher’s exact tests, four variables showed statistically significant differences. More participants in the HPP arm decreased their frequency of alcohol consumption (+22.7%, *p* < 0.05), increased their daily fruit consumption (+14.7%, *p* < 0.05), and increased their consumption of healthy fats (+31.8%, *p* < 0.001). In contrast, more participants in the GPN arm increased their daily consumption of fizzy drinks (+10.2%, *p* < 0.01). No other statistically significant changes were observed in the outcome measures between HPP and GPN groups.

## Discussion

### Summary of key findings

This study aimed to establish the likely clinical effectiveness of a pilot primary prevention programme for patients with high risk of cardiovascular risk in general practice (HRPP). This study’s findings show that the HRPP was much needed due to the high levels of health risk observed among patients, and that a future definitive HRPP trial, albeit one confounded by the COVID-19 pandemic, is most likely to be effective in terms of combatting health issues regarding weight, exercise, and diet. There is also promising evidence that definitive trials may be effective in terms of tackling problems relating to mental health, health motivation, cardiovascular risk knowledge, diet, triglyceride levels, detection of health issues (e.g. diabetes), smoking, and sedentary behaviour. Both the GPN and HPP arms yielded positive clinical outcomes, although the HPP arm showed better returns in terms of patient recruitment and promoting a healthier diet. Further, despite considerable logistical challenges imposed by the COVID-19 pandemic, the HRPP demonstrated good levels of acceptability. Recruitment and retention rates for a future definitive HRPP trial are likely to be high as good levels of engagement were evident from practices and patients across both GPN and HPP arms in the programme, its baseline and follow-up assessments.

### Comparison of findings with existing literature

The findings support existing evidence heralding the value of interventions promoting high risk patients’ capacity for health self-management [17], and particularly evidence highlighting the positive effect that such interventions can have regarding improvements in areas such as weight loss and health behaviour [18, 19]. The merits of interventions involving health promotion professionals with expertise in healthy lifestyle coaching, especially regarding improvements in patients’ diet and exercise, were evident in this study. These findings are novel as previous research highlighting the positive impact that such professionals can have in targeted CVD interventions is limited [20]. It was also notable that the findings depicting this study sample’s characteristics support previous assertions in the literature that elevated cardiovascular health risk is a concern in disadvantaged communities [2]. The findings also add fresh evidence demonstrating the degree of risk in disadvantaged Irish communities, and the promise of telemedicine CVD interventions arising during the COVID-19 pandemic [21].

### Methodological strengths & limitations

The study sample itself was a strength of this project. While previous research has examined the impact of cardiovascular health promotion interventions in deprived communities [2], no studies of this nature, at least to our knowledge, have demonstrated the likely clinical utility of such interventions in disadvantaged Irish areas. However, the absence of a control group comparing the HRPP intervention cohort with patients receiving usual care was a design limitation. The participating general practices facilitated the ability to gain fresh insight into the prevalence, demographic profiles, lifestyles, and health motivations of high-risk patients in the Ireland East region, and future research and clinical/public health initiatives in this region can be greatly informed by the project’s learnings. It is also worth noting that large parts of the HRPP coincided with the COVID-19 pandemic in Ireland. Studies have acknowledged familial premature cardiovascular disease (CVD) deaths as a significant risk factor in cardiovascular disease assessment [22, 23]. However, this particular risk factor was not incorporated into the pilot study. Its potential inclusion in future definitive trials could enhance the comprehensiveness of risk assessment protocols. The programme, participating practices, the GPNs and HPP as well as the lives of enrolled patients, were considerably disrupted by the pandemic, and so, delivering the programme and conducting its evaluation as originally planned was a major and unexpected challenge.

### Implications for future research, clinical practice, and policy

This study’s findings can play an important role in supporting future research in the area. A randomised controlled study with a larger sample comparing the HRPP intervention and usual care is needed to confirm the potential the HRPP compared to standard care delivery for high-risk patients. It is unclear if the COVID-19 pandemic altered patients’ attitudes to adherence to a health promotion intervention, so further research post pandemic is needed to assess adherence to the programme. The study’s findings further support the view that there is value in incorporating structured health promotion initiatives into routine practice. These programmes may be particularly beneficial if delivered by staff with strong health behaviour expertise and availability.

## Conclusion

This study showed that the HRPP was a much-needed intervention, that the programme has strong potential in the areas of improving high-risk patients’ cardiovascular related health and health behaviours, and that teams working in primary care are well suited to addressing CVD risk in communities. The findings may inform future chronic disease management strategy, particularly in primary care, and future definitive trials of the HRPP are recommended.
